# Effects of Myofascial Chain-Based Radial Extracorporeal Shock Wave Therapy in Chronic Non-Specific Low Back Pain: A Randomized Controlled Trial

**DOI:** 10.3390/jcm15218531

**Published:** 2026-11-02

**Authors:** InSeong Choi, JongEun Yim

**Affiliations:** Department of Physical Therapy, Graduate School, Sahmyook University, 815, Hwarang-ro, Nowon-gu, Seoul 01795, Republic of Korea; limited2022@gmail.com

**Keywords:** chronic non-specific low back pain, radial extracorporeal shock wave therapy, myofascial chain, fascia, randomized controlled trial

## Abstract

**Background**: Chronic non-specific low back pain (NSLBP) is a leading cause of disability worldwide. While radial extracorporeal shock wave therapy (rESWT) is commonly utilized, conventional protocols primarily target localized pain sites and may overlook global myofascial dysfunction. This study compared the clinical effectiveness of myofascial chain-based rESWT with conventional localized rESWT in patients with chronic NSLBP receiving standardized conservative therapy. **Methods**: In this prospective, assessor- and participant-blinded randomized controlled trial, 30 adults with chronic NSLBP were randomly assigned (1:1) to either a myofascial chain-based rESWT group (*n* = 15) or a localized rESWT group (*n* = 15). Both groups received four weekly sessions of active rESWT (3000 pulses/session) combined with identical physical therapy and home-based stabilization exercises. Primary outcomes were pain intensity (10-cm Visual Analog Scale, VAS) and functional disability (Oswestry Disability Index, ODI, 0–50 scale). The secondary outcome was lumbar flexion excursion assessed by the Modified Schober Test (MST). Assessments occurred at baseline (Week 0), immediately post-treatment (Week 5), and at 8-, 12-, and 16-week follow-ups, with the 16-week follow-up designated as the primary trial endpoint. Analyses were conducted based on the intention-to-treat (ITT) principle using repeated-measures ANOVA and ANCOVA. **Results**: All 30 participants were included in the primary ITT analysis. Both groups showed significant improvements over time across all outcomes (*p* < 0.001). Significant Group × Time interaction effects favored the myofascial chain group for VAS (*F* = 20.31, *p* < 0.001, *ηp*2 = 0.42) and ODI (*F* = 16.68, *p* < 0.001, *ηp*2 = 0.37). At the primary 16-week endpoint, repeated-measures model contrasts favored the myofascial chain group for VAS (mean difference: −0.65, 95% CI [−1.31 to 0.02], *p* = 0.057) and ODI (mean difference: −3.20, 95% CI [−5.62 to −0.78]). For lumbar mobility, ANCOVA adjusting for baseline imbalance demonstrated greater improvement in the myofascial chain group at 3 months (*F* = 5.54, *p* = 0.026, *ηp*2 = 0.170; adjusted mean difference: 0.19 cm, 95% CI [0.03 to 0.36]). Symptom recurrence did not significantly differ between groups (6.7% vs. 13.3%, *p* = 1.00). Expected mild procedure-related soreness and erythema were observed, but no severe treatment-related adverse events occurred. **Conclusions**: When added to standard rehabilitation, myofascial chain-based rESWT yielded greater reductions in pain and disability and better restoration of lumbar mobility than localized rESWT. Trial registration: Clinical Research Information Service (CRIS, KCT0011599; retrospectively registered on 13 February 2026). However, worst-case sensitivity analyses indicated that superiority at the 16-week endpoint was sensitive to missing data assumptions among participants who experienced recurrence. Therefore, these comparative findings should be interpreted cautiously as exploratory, and larger multicenter trials with higher retention rates are required to definitively establish long-term superiority.

## 1. Introduction

Low back pain (LBP) is the most prevalent musculoskeletal disorder worldwide and remains the leading cause of years lived with disability across all age groups. Beyond its substantial personal burden, LBP markedly reduces quality of life and imposes a high socioeconomic cost on healthcare systems globally [1]. Despite extensive research and diverse treatment strategies, LBP continues to present a major clinical challenge.

Approximately 85% of LBP cases are classified as non-specific low back pain (NSLBP), indicating the absence of a clearly identifiable pathological or neurological cause [2,3]. NSLBP is characterized by high prevalence and recurrence rates, with one-year prevalence reported to reach up to 73% and lifetime prevalence exceeding 70% in many industrialized countries [2,3]. Patients with chronic NSLBP frequently experience persistent pain, functional limitations, and reduced participation in daily activities, while long-term treatment outcomes remain suboptimal.

Conventional management of chronic NSLBP has relied heavily on maximum nonoperative therapies, including pharmacological interventions such as nonsteroidal anti-inflammatory drugs, muscle relaxants, opioid analgesics, and lumbar epidural steroid injections. The utilization and associated costs of these approaches have increased steadily over time, raising concerns regarding long-term effectiveness, safety, and healthcare sustainability [1,2]. Consequently, there is a growing demand for effective, non-invasive, and cost-efficient therapeutic alternatives.

Current clinical guidelines emphasize the integration of non-pharmacological strategies, including exercise therapy, manual therapy, and myofascial techniques, as core components of NSLBP management [4,5]. However, there remains a clear need for adjunctive physical modalities that can enhance the effects of conservative rehabilitation, particularly in patients with persistent or recurrent symptoms.

Extracorporeal shock wave therapy (ESWT) has emerged as a promising non-invasive intervention for a range of musculoskeletal pain conditions, including chronic low back pain [6,7]. ESWT delivers high-energy mechanical impulses to biological tissues, promoting local blood flow, mechanotransduction, and tissue regeneration, which collectively contribute to pain reduction and functional improvement [8]. While previous studies have demonstrated the general efficacy of ESWT in NSLBP, most protocols have focused on localized application to the site of pain [9,10].

A localized treatment strategy, although potentially effective for short-term symptom relief, may not adequately address the underlying mechanisms contributing to chronicity and recurrence in NSLBP. Increasing evidence suggests that NSLBP is associated with altered load transfer, global myofascial tension, and impaired movement coordination rather than isolated segmental dysfunction [11]. From this perspective, interventions targeting only the symptomatic lumbar region may fail to resolve systemic biomechanical imbalances.

The myofascial chain concept provides a theoretical framework that reflects the functional continuity of muscles and fascia throughout the body. Originating from early descriptions of muscle chains and later formalized through anatomical and biomechanical research, this concept proposes that tension generated in one region can be transmitted along interconnected myofascial pathways to influence distant structures [12,13,14,15]. Within this model, dysfunction in the lumbopelvic region may be both a source and a consequence of altered tension along posterior or lateral myofascial chains.

Contemporary rehabilitation paradigms grounded in biotensegrity further support the view of the musculoskeletal system as an integrated tension network rather than a collection of independent anatomical units. Accordingly, effective management of chronic NSLBP may require therapeutic strategies that address global myofascial continuity and systemic tension patterns, rather than solely focusing on local pain sites.

Although the clinical benefits of ESWT for NSLBP have been reported, investigations examining the efficacy of ESWT protocols explicitly based on the myofascial chain concept remain limited. To our knowledge, clinical trials directly comparing an explicit myofascial chain-based rESWT protocol with conventional localized lumbar rESWT in patients with chronic NSLBP remain scarce.

Therefore, the purpose of this randomized controlled trial was to evaluate the clinical efficacy of myofascial chain-based rESWT in patients with chronic NSLBP and to determine whether this approach provides superior improvements in pain intensity, functional disability, and lumbar range of motion compared with a localized rESWT protocol. We hypothesized that myofascial chain-based rESWT would result in greater improvements in pain, disability, and lumbar mobility than localized rESWT.

## 2. Materials and Methods

### 2.1. Study Design

Patient recruitment, intervention, and follow-up assessments were conducted from June 2025 to February 2026 at the manual therapy room of Green Hospital in Guri-si, Gyeonggi-do, Korea. This study was designed as a prospective, single-center, randomized controlled trial.

The study was conducted in accordance with the guidelines of the Declaration of Helsinki [16] and approved by the independent Bioethics Committee of Sahmyook University, Republic of Korea (approval no. SYU IRB 2023-04-009-007; initially approved on 19 April 2023). All participants provided written informed consent after receiving a thorough explanation of the study procedures and data confidentiality. Adhering to the CONSORT reporting standards, the trial was registered with the Clinical Research Information Service (CRIS, KCT0011599; registered on 13 February 2026). Due to administrative delays, registration was completed retrospectively following follow-up completion; however, the original IRB-approved study protocol, eligibility criteria, and pre-specified outcome measures were established prior to enrollment and remained unchanged throughout the trial (Supplementary File S1).

### 2.2. Participants

**Figure 1:**
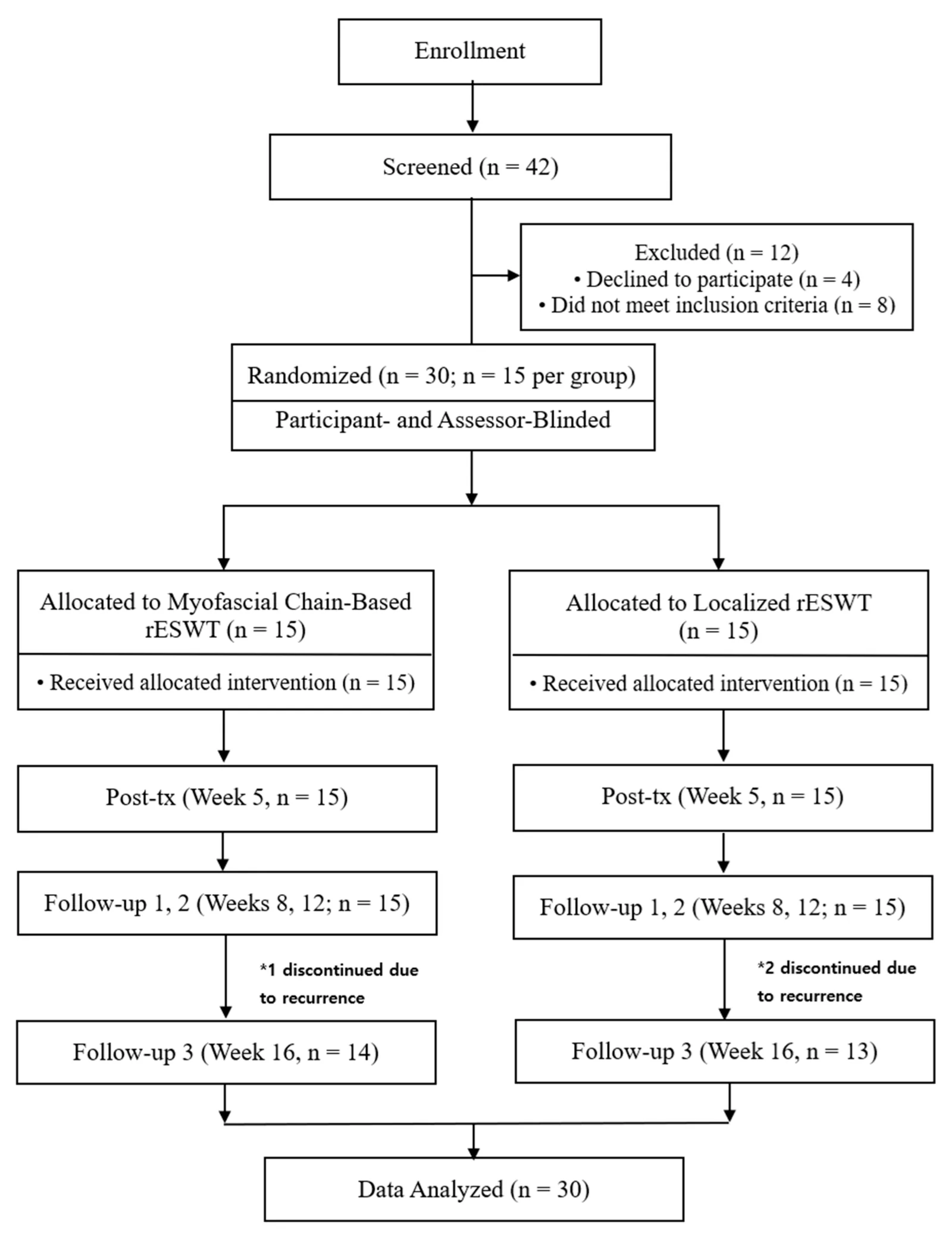
Consort flow chart for the patients through the study. * Indicates the number of participants who discontinued follow-up visits due to symptom recurrence.

A total of 42 consecutive outpatients with chronic low back pain were screened for eligibility at the Department of Rehabilitation Medicine between June 2025 and October 2025. Of these, 4 declined to participate and 8 were excluded for failing to meet the selection criteria; the remaining 30 participants (16 females, 14 males) provided written informed consent and were randomized (1:1). The detailed participant flow across all assessment visits is displayed in Figure 1.

Inclusion criteria were: (1) adults between 18 and 70 years of age; (2) non-specific LBP persisting for more than three months; (3) clinically diagnosed chronic non-specific LBP characterized by recurrent lumbosacral stiffness, swelling, or pain, confirmed by either (i) X-ray findings that excluded lumbar vertebral fracture, spondylolysis, spondylolisthesis, and severe osteoporosis, or (ii) normal findings on Magnetic Resonance Imaging (MRI) or the presence of low signal intensity disc herniation (defined as mild, asymptomatic bulging without neurological signs); and (4) submission of a personally signed and dated written informed consent form, indicating the patient’s willingness to participate in the study.

Exclusion criteria were: (1) individuals under 18 years or over 70 years of age; (2) unwillingness to participate or failure to personally sign and date the written informed consent form; (3) previous history of spinal fracture or spinal surgery; (4) specific spinal pathology or any symptomatic lumbar disc herniation/protrusion accompanied by clinical radiculopathy (confirmed by dermatomal sensory deficit, lower-extremity motor weakness, diminished tendon reflexes, or a positive straight-leg raise test < 60^∘^ adjudicated by a board-certified rehabilitation specialist), ankylosing spondylitis, structural scoliosis, spondylolisthesis, or spondylolysis; (5) presence of systemic or mental disorders; and (6) any contraindication to radial Extracorporeal Shock Wave Therapy (rESWT), including pregnancy, the presence of blood coagulation disorders (including local thrombosis), local tumor, local bacterial or viral infection (including lumbar tuberculosis), oral anticoagulant therapy, or local corticosteroid application within six weeks prior to the first rESWT session.

### 2.3. Randomization and Blinding

This study was designed as a prospective, participant- and assessor-blinded randomized controlled trial. Following baseline assessment, participants were randomly allocated to either the experimental or control group using a computer-generated random allocation sequence (https://www.random.org, accessed on 15 May 2025). To ensure allocation concealment, the sequence was managed using sequentially numbered, opaque, sealed envelopes stored in a double-locked cabinet by an independent research coordinator (Researcher A) who also verified eligibility consent but was uninvolved in assessment or intervention delivery.

A designated treating physical therapist (Therapist B) opened the assigned envelope sequentially only upon participant entry into the treatment suite and administered the rESWT interventions. Because of the distinct anatomical regions treated, Therapist B could not be blinded.

A dedicated clinical outcome assessor and data analyst (Assessor C) remained strictly masked to group allocation throughout all evaluations and database curation. Assessor C was physically segregated from the intervention area and was strictly prohibited from discussing intervention details with participants. Because both groups received active rESWT using identical device hardware, physical applicator contact, acoustic impulses, and prone positioning, participants were blinded to the study hypotheses and were unaware of which application strategy was hypothesized to be superior. However, complete procedural masking to the anatomical treatment sites was inherently impossible for the participants; thus, blinding was successfully directed at the study hypotheses rather than full procedural unawareness.

### 2.4. Interventions

The treatment protocols for the experimental and control groups were executed as follows:

#### 2.4.1. Radial Extracorporeal Shock Wave Therapy (rESWT) Protocol and Positioning

All rESWT procedures were performed using the ShockMaster 500 (GymnaUniphy NV, Bilzen, Belgium) device equipped with the D-actor 20 mm “T” applicator. For all applications, standard ultrasound gel was generously applied to the skin to ensure acoustic coupling, minimize tissue resistance, and facilitate smooth movement of the applicator.

Both groups underwent one session per week for four consecutive weeks (a total of four sessions). The operational parameters were standardized across both groups: 1.0 bar working pressure and a delivery frequency of 5–7 Hz [17]. The pressure was maintained at 1.0 bar throughout all sessions to prevent intolerable discomfort or procedure-induced muscle guarding that could compromise adherence. Each rESWT session delivered exactly 3000 pulses and lasted approximately 8 to 10 min (operating at 5–7 Hz). Patients were positioned prone on a standard treatment table with a supportive pillow placed under the lower abdomen to minimize lumbar lordosis and relax the paraspinal musculature. A total dosage of 3000 pulses per session was selected in accordance with established clinical consensus and prior clinical trials demonstrating optimal therapeutic mechanotransduction for chronic low back pain without inducing tissue microdamage [9,17].

#### 2.4.2. Experimental Group (Myofascial Chain-Based rESWT)

Participants in the experimental group received radial extracorporeal shock wave therapy (rESWT) applied according to established principles of myofascial continuity and force transmission. Treatment regions were selected based on anatomically and biomechanically interconnected structures implicated in lumbopelvic load transfer and postural control, as described in contemporary fascial research [18,19,20,21,22].

**Figure 2:**
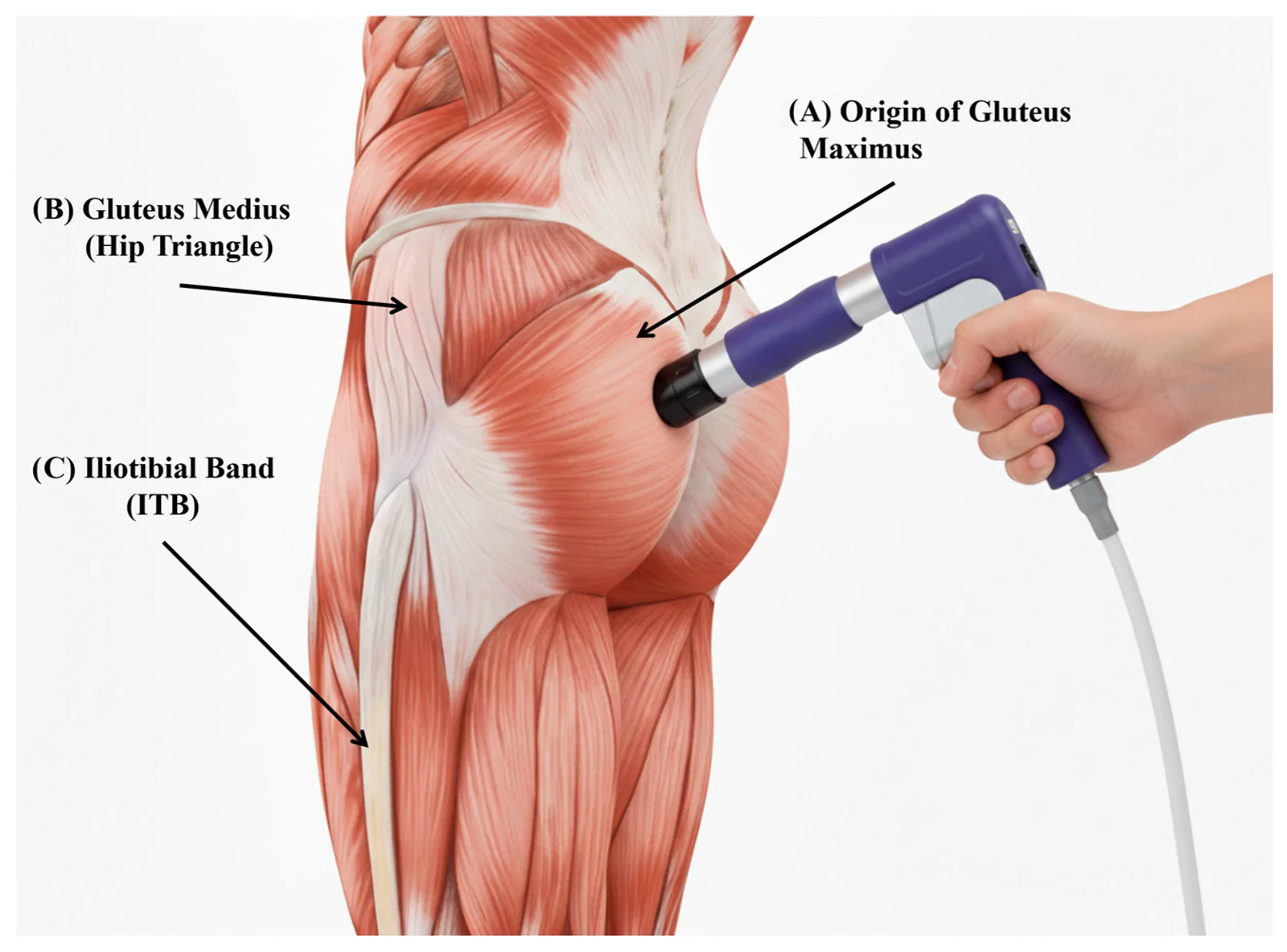
Myofascial chain-based rESWT protocol. Schematic illustration of the radial extracorporeal shock wave therapy (rESWT) application sites in the myofascial chain-based intervention. Treatment regions included the gluteus maximus (**A**), gluteus medius within the hip triangle (**B**), and iliotibial band (**C**), which are anatomically and functionally interconnected structures involved in lumbopelvic load transfer. These regions correspond to commonly described myofascial continuity pathways, including the Back Functional Line and Lateral Line, as summarized in the Anatomy Trains framework. The intervention targeted these interconnected regions to address global myofascial tension rather than isolated lumbar pain sites.

The selection of the gluteus maximus, gluteus medius, and iliotibial band corresponds to commonly described myofascial continuity models, including the Back Function Line and Lateral Line, which are summarized in the Anatomy Trains framework proposed by Myers [13] (Figure 2). However, the present intervention was grounded primarily in anatomical and biomechanical evidence demonstrating functional force transmission across fascial tissues rather than in adherence to a single conceptual model [18,19,20].

The gluteus maximus plays a key role in posterior load transfer through its fascial connections with the thoracolumbar fascia, contributing to lumbar spine stability and force distribution during functional movement [21]. The gluteus medius is a primary lateral stabilizer of the pelvis and has been shown to influence frontal-plane control and lumbar loading through its integration with lateral fascial structures [19,22]. The iliotibial band represents a major tensile element of the lateral fascial system and is involved in transmitting mechanical forces between the pelvis and lower extremity [18,20].

By sequentially applying rESWT to these interconnected regions, the experimental intervention aimed to modulate fascial stiffness, improve mechanical load sharing, and reduce abnormal tension patterns across the lumbopelvic–lower extremity complex, rather than focusing solely on localized lumbar pain sites.

A total of 3000 pulses were administered per session using a dynamic, circular moving application technique along the identified myofascial pathways. For unilateral symptoms, 1000 pulses were delivered sequentially to each of the three target sites: (1) the origin and muscle belly of the gluteus maximus, (2) the gluteus medius within the hip triangle, and (3) the proximal and mid-portion of the iliotibial band (ITB). In participants presenting with bilateral symptoms or central low back pain without clear lateralization, the 3000 pulses were evenly divided between both sides (500 pulses per site bilaterally, totaling 1500 pulses per side) to maintain equal systemic volume. The lumbar spine itself was intentionally omitted from shock wave application in this group to isolate the remote biomechanical effects of the myofascial chain protocol. All anatomical landmarks were palpated and confirmed prior to each session by a licensed physical therapist.

#### 2.4.3. Control Group (Localized Pain rESWT)

Patients in the control group received rESWT using identical device settings, coupling gel, and prone positioning. The therapy was administered using the same dynamic moving applicator technique to the two primary tender points identified by palpation and patient feedback within the lumbosacral region. A total of 3000 pulses were delivered per session (1500 pulses per tender point). In cases of bilateral symptoms, the two primary points were selected on the contralateral lumbar sides (1500 pulses per side), ensuring that the total energy delivered and contact duration were perfectly matched between groups. Application was strictly restricted to the symptomatic lumbar and sacral regions, with no shock waves delivered to the gluteal or thigh regions.

#### 2.4.4. Common Therapeutic Interventions and Standardization

To mirror routine clinical practice, both groups concurrently received a standardized conservative physical therapy regimen and a home-based stabilization program:

- Clinical Modalities: Immediately following each rESWT session, all participants received superficial heat therapy (hot pack for 20 min), therapeutic continuous ultrasound (1.0 MHz, 1.5 W/cm^2^ for 5 min), and conventional transcutaneous electrical nerve stimulation (TENS: symmetrical biphasic square pulses, 100 Hz, 100 μs, sensory intensity for 15 min). Subsequently, standardized myofascial release (gentle effleurage and petrissage) was applied to the bilateral lumbar erector spinae muscles for 5 min by a physical therapist.
- Home-Based Functional Training: All participants were instructed to perform a standardized lumbo-pelvic stabilization routine daily at home (at least 15 min per day). The program comprised four core exercises: abdominal drawing-in maneuvers in the supine hook-lying position, pelvic bridges, quadruped bird-dog progressions, and side-lying clamshells (each performed for 3 sets of 10 repetitions with a 5-s hold). Participants were provided with an illustrated instruction booklet and maintained a daily compliance logbook, which was reviewed weekly during clinical visits to quantify adherence. Across the 4-week active intervention phase (28 prescribed daily sessions), home-exercise adherence was 91.3% ± 6.4% in the experimental group and 89.8% ± 7.1% in the control group, with all 30 participants completing 100% of the four weekly in-clinic physical therapy sessions.

Throughout the trial, participants were instructed to record any intake of rescue analgesics (permitted only for unbearable acute flares, limited to acetaminophen up to 1000 mg/day). Receiving concurrent spinal injections, manual manipulation, or outside physiotherapy was strictly prohibited as a protocol violation.

### 2.5. Outcome Measures

#### 2.5.1. Primary Outcomes

The primary outcomes of this study were pain intensity and functional disability.

Pain intensity was assessed using a 10-cm horizontal Visual Analog Scale (VAS). Participants placed a vertical mark on a continuous 100-mm line representing their average low back pain over the preceding 24 h, ranging from 0 (“no pain”) to 10 (“worst imaginable pain”), measured to the nearest 1 mm (0.1 score precision).

Functional disability was evaluated using the validated Korean version of the Oswestry Disability Index (ODI). The total ODI score was analyzed as a continuous raw score ranging from 0 to 50 points (the direct sum of the 10 individual sections scored 0–5). In the event of unanswered sections, standard proportional scoring is recommended; however, all participants in this trial completed 100% of questionnaire items without missing responses. The primary trial endpoint was prospectively designated as the 16-week assessment (3 months post-intervention). A pre-specified decision rule required both primary outcomes (VAS and ODI) to demonstrate statistically significant between-group differences to support overall superior efficacy.

#### 2.5.2. Secondary Outcome

The secondary outcome of this study was lumbar spine range of motion (ROM).

Lumbar spine mobility was assessed using the Modified Schober Test (MST), reflecting lumbar flexion excursion. The test was performed by marking a primary point at the lumbosacral junction (L5 level), then marking 5 cm inferiorly and 10 cm superiorly (total initial distance of 15 cm). Patients were instructed to flex their trunk maximally while keeping their knees straight. The new distance between the top and bottom marks was measured, and the recorded value represents the excursion (i.e., new distance minus 15 cm). Measurements were recorded to the nearest 0.1 cm.

All assessments were conducted across five standardized elapsed-time milestones relative to study inception: Baseline (Week 0, immediately prior to Session 1), Post-treatment (Week 5, 1 week following Session 4), Follow-up 1 (Week 8, 4 weeks post-treatment), Follow-up 2 (Week 12, 8 weeks post-treatment), and Follow-up 3/Primary Endpoint (Week 16, 12 weeks post-treatment).

A structured overview of all primary and secondary outcome measures—including their evaluated domains, scoring ranges, assessment schedules, and established minimal clinically important difference (MCID) thresholds—is summarized in Table 1 (Table summary of outcome measures).

### 2.6. Sample Size

The sample size was determined a priori using G*Power (version 3.1.9.7) for a repeated-measures ANOVA (within-between interaction model). Based on previous rESWT trials evaluating pain and disability in chronic low back pain [9], an effect size *f* = 0.40 (Cohen’s benchmark for a large effect, corresponding to $\eta_{p}^{2}\approx 0.14$) was specified. With a two-tailed alpha of 0.05, desired statistical power (1 − *β*) of 0.80, two groups, five measurement time points, an assumed correlation among repeated measures of *r* = 0.50, and a nonsphericity correction factor *ϵ* = 0.70, the minimum required sample size was 24 participants (12 per group). To accommodate an anticipated 20% attrition rate during the longitudinal follow-up period, the final recruitment target was set at 30 participants (15 per group).

### 2.7. Statistical Analysis

Baseline demographic variables were compared between the treatment groups using the independent *t*-test or the Mann–Whitney U test for continuous data, and the Chi-square test of independence for categorical data. Normality of data distribution was assessed using the Shapiro–Wilk test.

Comparisons of outcomes obtained before and after treatment with rESWT, as well as at the follow-up 2 (Week 12) and follow-up 3 (Week 16), were conducted using Repeated Measures Analysis of Variance (ANOVA) followed by post-hoc tests (specifically, Bonferroni correction). Additionally, Two-Way Repeated Measures ANOVA was utilized to analyze the effects of the two factors (time and group) on the outcomes and their interaction. Greenhouse–Geisser correction was applied when the assumption of sphericity was violated.

Because a statistically significant baseline difference was observed between groups in lumbar range of motion assessed by the Modified Schober Test (MST), an Analysis of Covariance (ANCOVA) was additionally performed to adjust for this imbalance. Baseline MST values were entered as a covariate, and group allocation was treated as the fixed factor to evaluate between-group differences in MST at the 3-month follow-up. Partial eta squared (*η*^2^) was calculated to estimate the effect size of the adjusted treatment effect.

Longitudinal outcome trajectories were evaluated using a repeated-measures General Linear Model (GLM). The model included ‘Group’ as a between-subjects factor and ‘Time’ as a within-subjects factor. Pairwise between-group contrasts at specific time points (e.g., Week 16) were derived from the estimated marginal means using the pooled residual error variance of the omnibus model, with Bonferroni adjustments applied strictly across the two groups within each visit to control for multiple comparisons (Supplementary File S2).

The LOCF imputation and the designation of Week 16 as the primary endpoint were established in the statistical analysis plan prior to data unblinding. To address the outcome-dependent attrition limitations of LOCF (Lachin, 2016) [24], a worst-case Baseline Observation Carried Forward (BOCF) sensitivity analysis was conducted for participants withdrawing due to recurrence.

All statistical analyses were performed using IBM SPSS for Windows version 22.0 (IBM Corp., Armonk, NY, USA). The level of statistical significance was set at α = 0.05.

## 3. Results

### 3.1. Baseline Characteristics and Homogeneity

Baseline demographic and clinical characteristics were comparable between the two groups, with no significant differences observed in age, sex distribution, pain duration, baseline VAS, or ODI scores (*p* > 0.05) (Table 2). A small but statistically significant difference was identified in baseline lumbar range of motion assessed by the Modified Schober Test (MST), with the experimental group demonstrating slightly greater baseline mobility (*p* < 0.05). Consequently, baseline MST was treated as a covariate in subsequent analyses.

### 3.2. Primary Outcomes: Pain Intensity and Functional Disability

Both groups demonstrated significant reductions in pain intensity (VAS) and functional disability (ODI) over time (main effect of time: *p* < 0.001 for both outcomes). However, the experimental group exhibited significantly greater improvements compared to the control group (Table 3).

**Figure 3:**
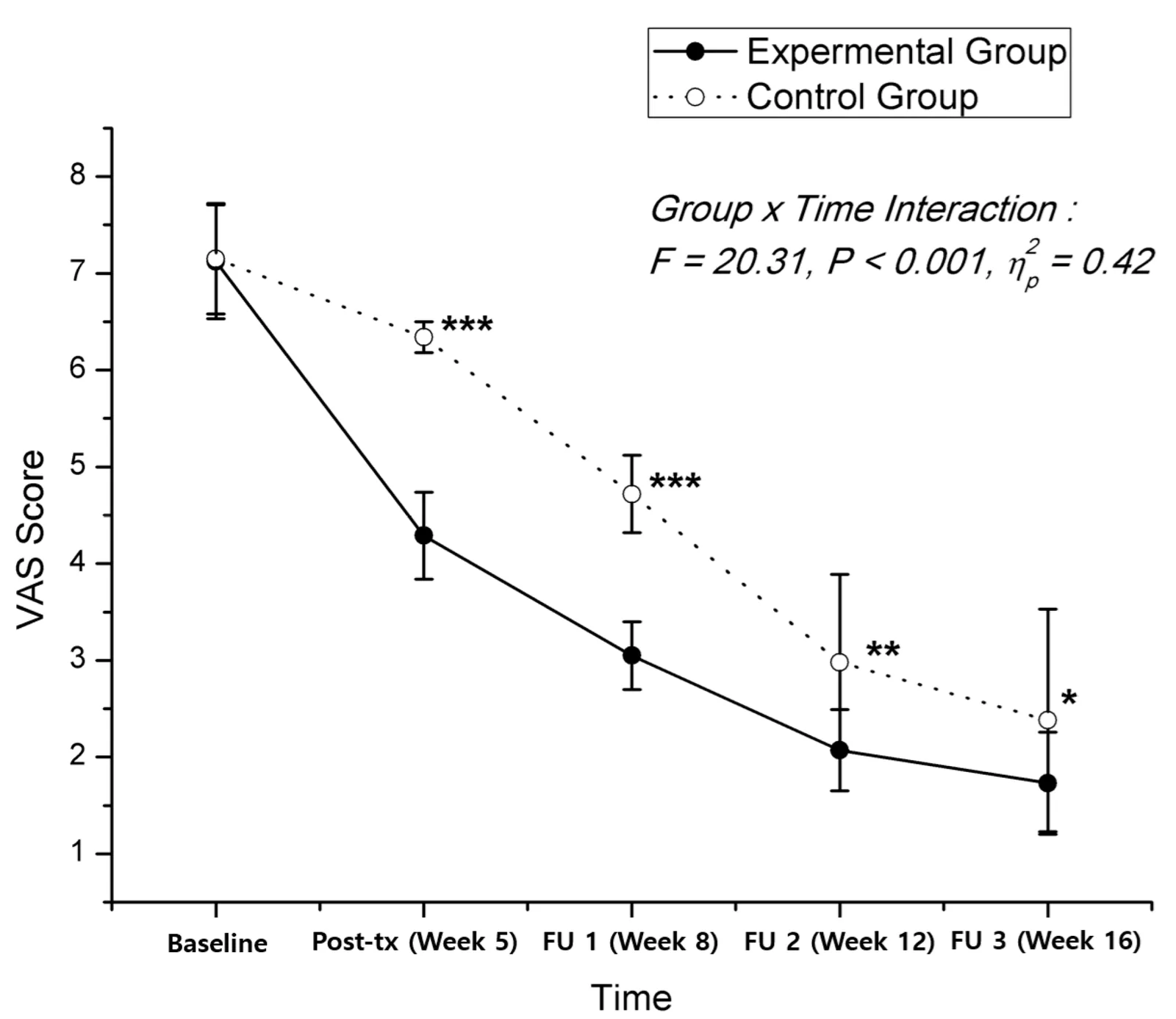
Comparison of longitudinal changes in pain intensity. Data are presented as mean ± standard deviation (SD) based on the intention-to-treat population (*N* = 30, LOCF imputation). The experimental group received myofascial chain-based radial extracorporeal shock wave therapy (rESWT), while the control group received localized rESWT. Lower Visual Analog Scale (VAS) scores indicate reduced pain intensity. Significant Group × Time interaction was observed (*F* (2.08, 58.10) = 20.31, *p* < 0.001, *ηp*2 = 0.42). p * <0.05, p ** <0.01, *** p<0.001 vs. Control group at the corresponding time point by post hoc pairwise comparisons.

For pain intensity, a significant Group × Time interaction effect was observed (*F* = 20.31, *p* < 0.001, *η*^2^ = 0.42), indicating a differential pattern of pain reduction favoring the myofascial chain-based rESWT group (Table 4, Figure 3). Post hoc analyses revealed that at the final follow-up 3 (Week 16), the experimental group demonstrated a lower VAS score compared to the control group, although this absolute difference at the specific Week 16 timepoint was marginally significant (mean between-group difference = −0.65, 95% CI [−1.31 to 0.02], *p* = 0.057).

**Figure 4:**
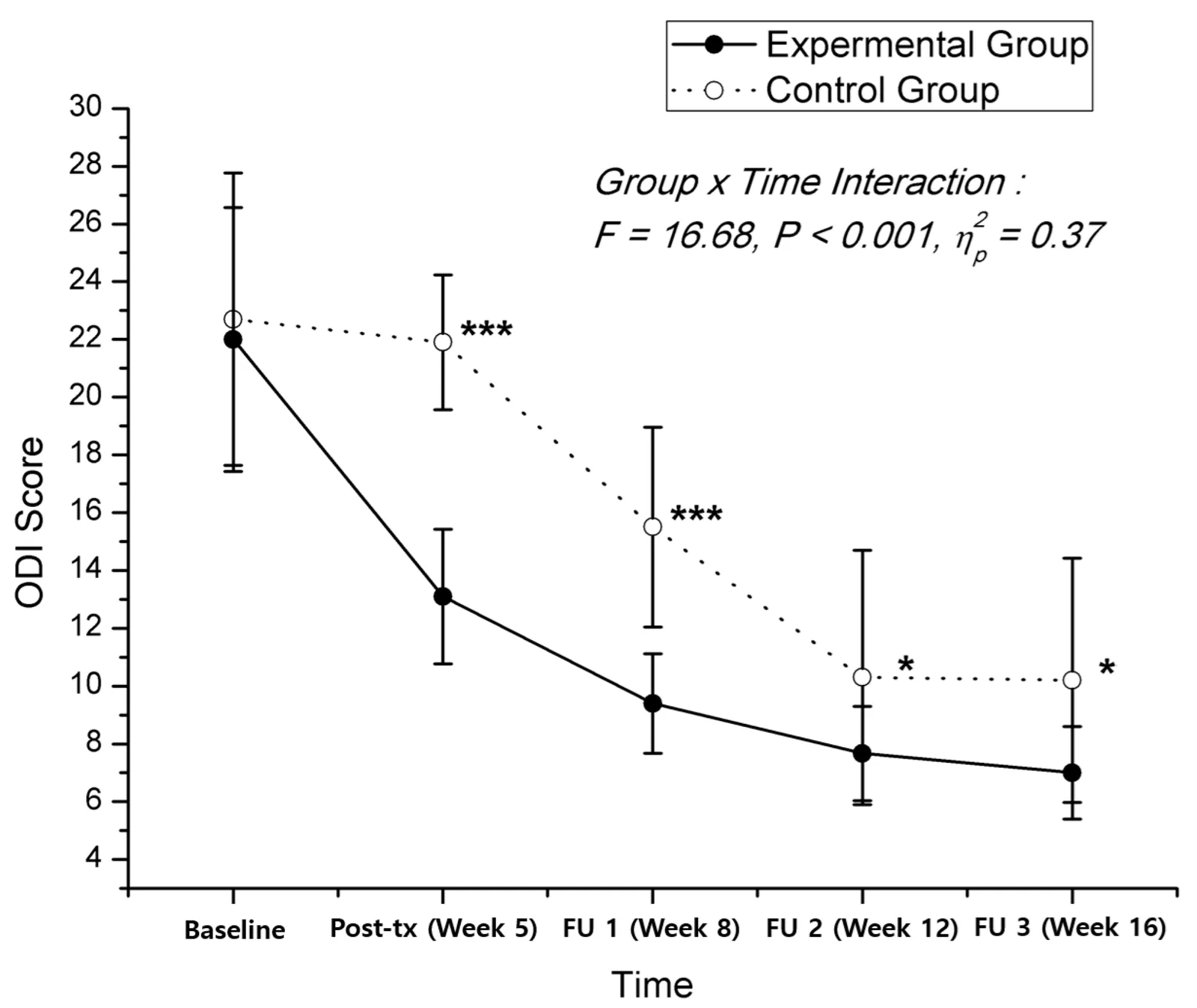
Improvements in functional disability measured by the Oswestry Disability Index (ODI). Longitudinal changes in mean ODI scores (0–50 raw scale). Error bars represent standard deviation (SD) of the ITT population with LOCF imputation. Both groups exhibited functional recovery; however, the myofascial chain-based approach resulted in significantly greater functional improvement across time (*F* (2.03, 56.84) = 16.68, *p* < 0.001, *ηp*2 = 0.37).  p *<0.05, *** p<0.001 vs. Control group at the corresponding time point.

Similarly, functional disability measured by the ODI showed a significant Group × Time interaction (*F* = 16.68, *p* < 0.001, *η*^2^ = 0.37) (Table 4, Figure 4). At follow-up 3 (Week 16), the experimental group demonstrated significantly greater functional improvement than the control group (mean between-group difference = −3.20, 95% CI [−5.62 to −0.78]).

### 3.3. Secondary Outcome: Lumbar Range of Motion

**Figure 5:**
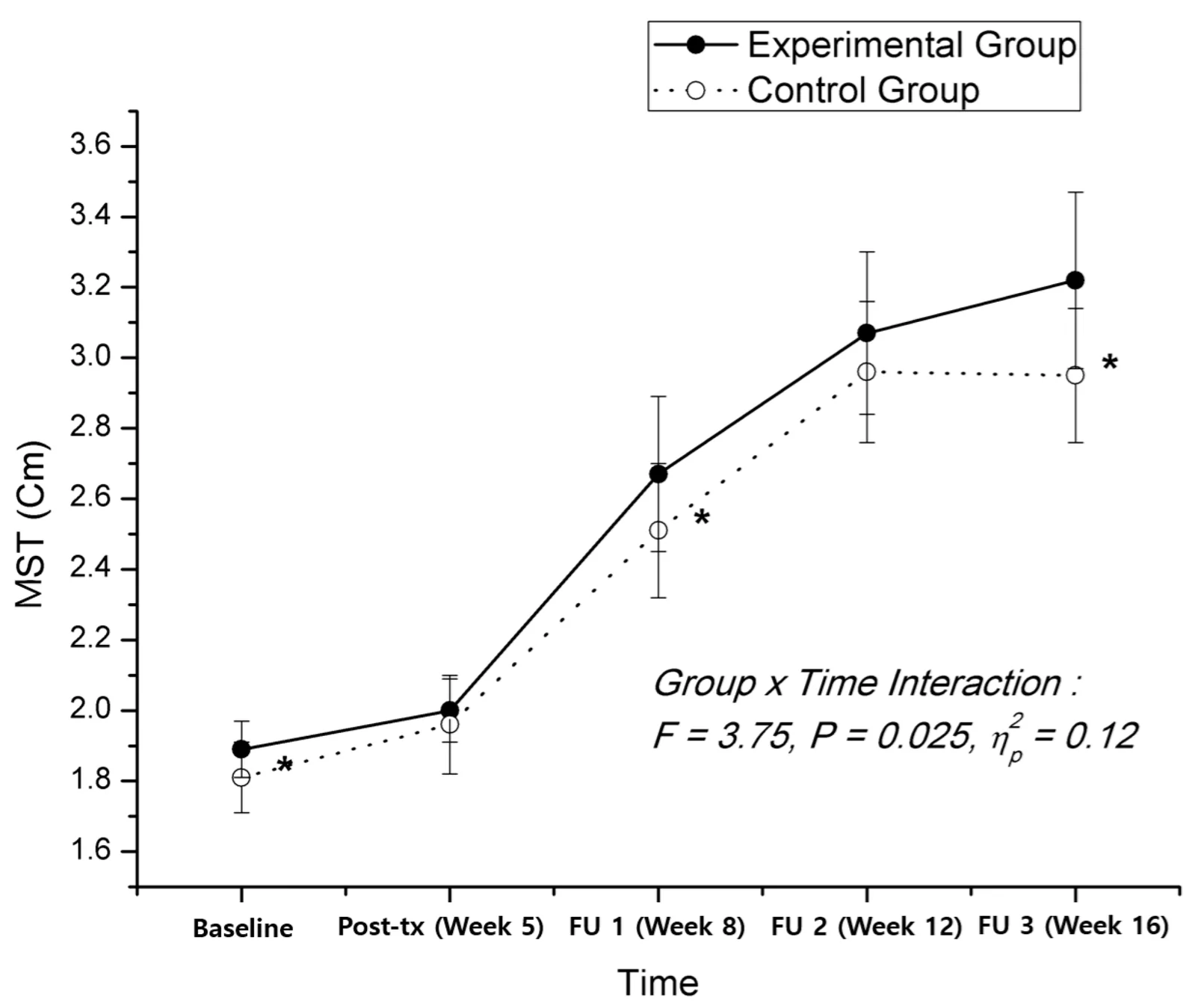
Enhancement of lumbar range of motion assessed by the Modified Schober Test (MST). Values represent mean ± standard deviation (SD) of lumbar flexion excursion (cm). Greater values indicate improved spinal flexibility. A statistically significant Group × Time interaction was observed ($F\;\left(2.22,62.18\right)=3.75,\;p=0.025,\;\eta_{p}^{2}=0.12$). At Follow-up 3 (Week 16), an Analysis of Covariance (ANCOVA) adjusting for baseline imbalance demonstrated a statistically significant treatment effect favoring the experimental group ($F\;\left(1,27\right)=5.54,\;p=0.026,\;\eta_{p}^{2}=0.170$; adjusted mean difference: 0.19 cm, 95% CI [0.03 to 0.36]). Unadjusted pointwise comparisons are denoted separately from the ANCOVA-adjusted endpoint.  p *<0.05 vs. Control group (unadjusted pairwise comparison at Post-tx [Week 5] and baseline-adjusted ANCOVA at Follow-up 3 [Week 16]).

Lumbar flexion mobility assessed by the MST increased significantly over time in both groups (main effect of time: Greenhouse–Geisser corrected $F\;(2.22,\;62.18)\;=\;606.47,\;p\;<\;0.001,\;\eta_{p}^{2}=0.96$) (Table 4). Furthermore, a statistically significant Group × Time interaction was observed, indicating a differential rate of mobility improvement between the two interventions (Mauchly’s test of sphericity *W* = 0.086, *p* < 0.001; Greenhouse–Geisser *ϵ* = 0.555; corrected $F\;(2.22,\;62.18)\;=\;3.75,\;p\;=\;0.025,\;\eta_{p}^{2}=0.12$) (Table 4, Figure 5). The overall between-group difference across time points was also statistically significant (*F* (1, 28) = 7.11, *p* = 0.013, *ηp*2 = 0.20).

Given the baseline imbalance in MST (*p* = 0.022), an analysis of covariance (ANCOVA) was performed using baseline MST as a covariate to evaluate the treatment effect at the 3-month follow-up. Even after adjusting for the baseline values using ANCOVA, the experimental group demonstrated significantly higher MST scores compared to the control group at follow-up 3 (Week 16) (*F* = 5.54, *p* = 0.026, *η*^2^ = 0.170), with an adjusted mean between-group difference of 0.19 cm (95% CI [0.03 to 0.36]) (Table 5). Although baseline MST significantly influenced the outcomes (*F* = 5.85, *p* = 0.023), these findings indicate that the experimental treatment provided an independent and significant contribution to the improvement of lumbar mobility beyond the influence of initial flexibility.

### 3.4. Attrition, Recurrence, Sensitivity, and Harms Analyses

All 30 randomized participants completed the 4-week active intervention phase as well as the Week 5 and Week 8 assessments (observed *n* = 15 per group). At Week 12, observed data remained complete for all 30 participants. Between Week 12 and Week 16, 1 participant in the experimental group (6.7%) and 2 participants in the control group (13.3%) experienced symptom recurrence. In accordance with the approved study protocol and patient information sheet, these 3 participants ceased physical attendance at scheduled follow-up visits to seek outside rescue treatments (specifically, oral nonsteroidal anti-inflammatory drugs and muscle relaxants prescribed at external clinics). However, they explicitly permitted the retention and scientific analysis of their previously collected data; formal consent for data utilization was not withdrawn (observed Week 16 denominators: *n* = 14 experimental, *n* = 13 control).

In this trial, symptom recurrence was operationally defined as an exacerbation of low back pain exceeding ≥2.0 points on the VAS persisting for >48 h that prompted the participant to seek outside pharmacological or medical intervention, occurring after achieving an initial ≥30% pain reduction during the 4-week treatment block. Recurrence events were confirmed through telephone interviews and medical chart documentation. In exploratory categorical analysis, recurrence rates did not differ significantly between groups (1/15 [6.7%] vs. 2/15 [13.3%], *p* = 1.00, two-sided Fisher’s exact test) (Table 6).

In the complete-case per-protocol sensitivity analysis (*N* = 27), between-group differences at Week 16 remained statistically significant and consistent with the primary ITT model: the VAS mean difference was −0.68 points (95% CI [−1.22 to −0.14], *p* = 0.015), and the ODI mean difference was −3.35 points (95% CI [−5.95 to −0.75], *p* = 0.013). However, under the worst-case Baseline Observation Carried Forward (BOCF) sensitivity imputation (wherein participants discontinuing due to recurrence were imputed with their pre-treatment baseline scores), the between-group differences at the primary Week 16 endpoint lost statistical significance for both VAS (mean difference: −0.32 points, 95% CI [−1.50 to 0.86], *p* = 0.583) and ODI (mean difference: −2.17 points, 95% CI [−6.16 to 1.82], *p* = 0.275). While the overall longitudinal Group × Time interactions remained significant across the entire 16-week study trajectory (*p* < 0.001 for both outcomes), absolute statistical superiority at the specific Week 16 endpoint was sensitive to worst-case missing data assumptions.

Regarding procedural harms, anticipated transient effects—such as mild procedure-related soreness and temporary localized erythema—were observed in 5 participants across both groups during the active treatment phase, but all symptoms resolved spontaneously within 24 h without medical intervention. No severe treatment-related adverse events, such as hematoma, bruising, skin burns, or neurological deterioration, were reported in either group throughout the study duration.

## 4. Discussion

### 4.1. Principal Findings

This randomized controlled trial investigated the clinical effectiveness of radial extracorporeal shock wave therapy (rESWT) applied according to the myofascial chain concept compared with conventional localized rESWT in patients with chronic non-specific low back pain (NSLBP). The most important finding of this trial is that the pattern and magnitude of clinical improvement differed meaningfully between the two rESWT application strategies. Because both groups received concurrent physical therapy and home-exercise programs, these findings highlight the relative advantage of targeting myofascial continuities rather than establishing the absolute independent efficacy of rESWT. Chronic NSLBP is widely recognized as a multifactorial condition characterized by persistent pain, functional disability, and high recurrence rates in the absence of specific structural pathology [25,26].

Specifically, both groups demonstrated significant time-dependent improvements in pain intensity (VAS), functional disability (ODI), and lumbar mobility (MST) (all *p* < 0.001), consistent with previous reports on ESWT in chronic low back pain [9,10,27]. However, the myofascial chain-based rESWT produced superior outcomes in pain reduction and functional recovery, as evidenced by significant Group × Time interaction effects for VAS (*F* = 20.31, *p* < 0.001, partial *η*^2^ = 0.42) and ODI (*F* = 16.68, *p* < 0.001, partial *η*^2^ = 0.37). In addition, after controlling for baseline differences in lumbar mobility, the myofascial chain approach exerted an independent and statistically significant effect on MST (ANCOVA: *F* = 5.54, *p* = 0.026, partial *η*^2^ = 0.170).

### 4.2. Pain Reduction and Functional Recovery

Pain intensity and disability are the primary drivers of healthcare utilization and reduced quality of life in patients with chronic NSLBP [25,28]. In the present study, the experimental group showed a marked reduction in VAS scores from 7.12 ± 0.59 at baseline to 1.73 ± 0.53 at the 3-month follow-up, whereas the control group improved from 7.15 ± 0.57 to 2.38 ± 1.15 over the same period. Although both trajectories reflect clinically meaningful improvement, the greater and more consistent reduction observed in the experimental group explains the large interaction effect size.

A similar pattern was observed for functional disability. The ODI score in the experimental group decreased from 22.00 ± 4.57 at baseline to 7.00 ± 1.60 at 3 months, while the control group improved from 22.73 ± 5.06 to 10.20 ± 4.23. From a clinical standpoint based on the 0–50 raw score system, this difference is substantial: the experimental group, on average, transitioned from severe disability (baseline) to minimal disability (3 months), whereas the control group remained closer to the threshold between minimal and moderate functional limitation.

These findings support the clinical rationale that treating NSLBP solely at the point of pain may be insufficient in many chronic cases. The myofascial chain concept emphasizes the anatomical and functional continuity of muscles and fascia as a force-transmitting system [18,19,20]. Anatomical and biomechanical studies have demonstrated that dysfunction in one segment of a myofascial chain can influence pain and movement in distant regions, supporting a more global treatment strategy [21,22].

Although the myofascial chain concept is frequently associated with the *Anatomy Trains* model proposed by Myers, it should be interpreted as a conceptual framework reflecting anatomical and biomechanical continuity of fascial tissues rather than as a discrete or universally accepted anatomical structure [18,19,20]. Contemporary anatomical and experimental studies have demonstrated the capacity of fascial tissues to transmit force across regions, providing a plausible mechanistic basis for the observed treatment effects.

When interpreting the clinical significance of these findings, it is important to consider the Minimum Clinically Important Difference (MCID). Established MCID thresholds (e.g., a 1.5–2.0 point reduction on the 10-cm VAS and a 5-point reduction on the 0–50 raw ODI scale; Ostelo et al., 2008) [23] are primarily designed to evaluate within-patient individual change rather than universal thresholds for between-group mean differences. In our study, both the experimental and control groups demonstrated substantial within-group improvements from baseline to Week 16 that exceeded these individual-level MCID thresholds (mean VAS reductions of 5.39 vs. 4.77 points; mean ODI reductions of 15.00 vs. 12.53 points, respectively). Regarding between-group comparisons, the incremental ODI difference at Week 16 (−3.20 raw points, 95% CI [−5.62 to −0.78]) highlights an additive comparative advantage favoring the myofascial chain approach. Notably, the confidence interval spans a range of benefit that encompasses clinically meaningful magnitudes, underscoring the potential value of targeting myofascial continuities when combined with active conservative rehabilitation.

### 4.3. Lumbar Mobility and the Role of ANCOVA

Lumbar mobility, assessed using the Modified Schober Test (MST), improved significantly over time in both groups, consistent with prior ESWT studies targeting spinal flexibility and soft tissue extensibility [10,27]. At baseline, a small but statistically significant difference was present between groups (experimental: 1.89 ± 0.08 cm; control: 1.81 ± 0.10 cm; *p* = 0.022). Given the clinical relevance of baseline flexibility, an ANCOVA was appropriately conducted with baseline MST as a covariate.

After adjustment, group allocation remained a significant predictor of post-treatment MST (*F* = 5.54, *p* = 0.026, partial *η*^2^ = 0.170), while baseline MST itself also exerted a significant influence (*F* = 5.85, *p* = 0.023). From a biomechanical and clinical standpoint, this result is noteworthy: it indicates that the superior gains in lumbar mobility observed in the experimental group were not merely a function of initial baseline differences, but rather reflected an independent treatment effect attributable to the myofascial chain-based intervention. This finding aligns with evidence that myofascial interventions can induce remote improvements in range of motion through force transmission along fascial continuities [29].

Clinically, the experimental group demonstrated a progressive increase in MST from 1.89 ± 0.08 cm at baseline to 3.22 ± 0.25 cm at 3 months, compared with an increase from 1.81 ± 0.10 cm to 2.95 ± 0.19 cm in the control group. This greater restoration of lumbar flexion excursion demonstrates improved gross spinal mobility; however, because localized fascial stiffness and intersegmental shear strain were not directly quantified using elastography or dynamic biomechanical imaging, fascial normalization remains a plausible working hypothesis rather than an established physiological mechanism.

### 4.4. Clinical Interpretation of the Myofascial Chain Approach

Chronic NSLBP is rarely the result of isolated tissue pathology. Instead, it reflects altered load transfer, persistent myofascial tension, and maladaptive movement strategies across the lumbopelvic region [26,28]. The myofascial chain-based rESWT protocol is consistent with contemporary models such as biotensegrity and fascial continuity, which conceptualize the musculoskeletal system as an integrated tension network rather than a collection of independent parts [18,19].

By applying rESWT along key components of the myofascial chain, the experimental intervention may have influenced not only local pain modulation but also remote tissue properties, such as fascial tension and neuromuscular coordination [8,9]. We hypothesize that when this mechanical stimulus is delivered along a myofascial continuum, it potentially facilitates more effective force transmission and intersegmental motion compared to localized treatments.

### 4.5. Recurrence and Sustainability of Outcomes

Recurrence remains a major challenge in the management of chronic NSLBP [30]. In the present study, symptom recurrence occurred in 1 participant (6.7%) in the experimental group and 2 participants (13.3%) in the control group, with no statistically significant difference between the two groups (*p* = 1.00, two-sided Fisher’s exact test). Given the small pilot sample size and the extremely low event count, this finding must be interpreted strictly as exploratory rather than confirmatory evidence of preventive efficacy.

Interventions that normalize biomechanical load distribution and restore functional movement patterns across myofascial continuities may theoretically offer a more durable therapeutic effect. However, because our statistical comparison was inevitably underpowered, definitive conclusions regarding long-term recurrence prevention cannot be drawn from the present trial. Future large-scale longitudinal investigations with extended follow-up windows are needed to determine whether myofascial chain-based rESWT genuinely confers superior protection against symptom recurrence.

### 4.6. Limitations and Future Directions

Several limitations should be acknowledged. The relatively small sample size limits generalizability and statistical power, particularly for recurrence analysis. In addition, the follow-up period was limited to three months, which restricts conclusions regarding long-term sustainability. Although ANCOVA was used to control for baseline differences in lumbar mobility, other factors such as psychosocial variables, motor control strategies, and occupational loading were not directly assessed [28,30].

Future studies should involve larger, multicenter cohorts with extended follow-up and incorporate objective assessments of fascial and neuromuscular properties, such as ultrasound elastography or motion analysis, to further elucidate the mechanisms by which myofascial chain-based rESWT exerts its clinical effects [17,29].

Additionally, retrospective registry registration, the inherent lack of participant masking to anatomical treatment sites, and the potential limitations of LOCF imputation under outcome-dependent missingness (as evidenced by the sensitivity of the Week 16 endpoint under worst-case BOCF assumptions) represent methodological constraints.

### 4.7. Clinical Implications

The present findings suggest that radial extracorporeal shock wave therapy targeting myofascial pathways provides an incremental benefit over localized application when embedded within multimodal conservative rehabilitation. From a clinical perspective, adopting a myofascial chain approach may help clinicians optimize movement efficiency in patients with chronic NSLBP. However, these comparative results should be viewed as preliminary, and routine implementation across general spine care must await confirmation from larger multicenter pragmatic trials.

## 5. Conclusions

When added to a standard rehabilitation program, myofascial chain-based rESWT provided superior improvements in pain, functional disability, and lumbar mobility compared with localized rESWT in patients with chronic NSLBP. These findings suggest that addressing global myofascial tension pathways may offer an incremental therapeutic advantage over treating isolated pain sites. However, worst-case sensitivity analyses (BOCF) revealed that the superiority at the primary 16-week endpoint was sensitive to missing data assumptions stemming from symptom recurrences. Given this lack of robustness under worst-case scenarios, along with the small pilot sample size and multimodal treatment setting, these findings must be interpreted strictly as exploratory. Future large-scale pragmatic trials with extended follow-up and higher retention rates are required to definitively confirm the underlying mechanistic effects and the long-term durability of the myofascial approach.

## Figures and Tables

**Table 1:** Summary of outcome measures, domains, measurement specifications, and MCID thresholds.

Outcome Measure	Domain/ Construct	Instrument/ Procedure	Scale & Range	Established MCID Threshold	Assessment Time Points
VAS (Primary)	Pain intensity	10-cm continuous horizontal visual analog line (recall period: past 24 h)	0–10 score (0: no pain, 10: worst pain imaginable)	1.5–2.0 points or ≥30% reduction	Baseline (W0), Post-tx (W5), Follow-up 1 (W8), Follow-up 2 (W12), Follow-up 3 (W16)
ODI (Primary)	Functional disability	Korean version of Oswestry Disability Index (10 sections)	0–50 raw score (0: no disability, 50: bed-bound/maximum disability)	5 points on the 0–50 raw scale (corresponding to 10 points or ≥30% reduction on the 0–100% scale; Ostelo et al., 2008) [23]	Baseline (W0), Post-tx (W5), Follow-up 1 (W8), Follow-up 2 (W12), Follow-up 3 (W16)
MST (Secondary)	Lumbar flexion mobility (excursion)	Modified Schober Test: 15 cm baseline span (5 cm inferior and 10 cm superior from L5/S1 junction); measured at maximum forward flexion	Continuous (cm) Precision: nearest 0.1 cm (recorded value = total flexed distance minus 15 cm)	Not established for non-inflammatory LBP (exploratory mobility metric)	Baseline (W0), Post-tx (W5), Follow-up 1 (W8), Follow-up 2 (W12), Follow-up 3 (W16)

Abbreviations: VAS, Visual Analog Scale; ODI, Oswestry Disability Index; MST, Modified Schober Test; MCID, Minimal Clinically Important Difference; FU, Follow-up.

**Table 2:** Baseline demographic and clinical characteristics of participants (*N* = 30).

Variable	Experimental (*n* = 15)	Control (*n* = 15)	*t*/*χ*^2^	*p*-Value
Age (years)	47.9 ± 15.02	47.8 ± 16.03	0.024	0.981
Sex (M/F)	7/8	7/8	*χ*^2^ = 0.00	1.000
Weight (kg)	70.3 ± 18.00	70.4 ± 12.12	−0.012	0.991
Height (cm)	166.6 ± 11.72	167.4 ± 8.94	−0.210	0.835
BMI (kg/m^2^)	25.1 ± 4.06	25.0 ± 3.09	0.051	0.960
Pain duration (months)	7.2 ± 1.90	7.4 ± 2.03	−0.279	0.782
VAS (Score)	7.12 ± 0.59	7.15 ± 0.57	−0.156	0.877
ODI (Score)	22.00 ± 4.57	22.73 ± 5.06	−0.417	0.680
MST (cm)	1.89 ± 0.08	1.81 ± 0.10	2.435	0.022 *

Values are mean ± SD. Independent *t*-tests and Chi-square tests were used. * *p* < 0.05.

**Table 3:** Changes in outcome measures over time (Intention-to-Treat, ITT).

Experimental Group (Myofascial Chain-Based rESWT)							
Outcome	Baseline (W0)	Post-tx (W5)	Follow-up 1 (W8)	Follow-up 2 (W12)	Follow-up 3 (W16)	*F*	*p*
VAS (Score)	7.12 ± 0.59 ^a^	4.29 ± 0.45 ^b^	3.05 ± 0.35 ^c^	2.07 ± 0.42 ^d^	1.73 ± 0.53 ^e^	373.09	<0.001 ***
ODI (Score)	22.0 ± 4.57 ^a^	13.1 ± 2.33 ^b^	9.40 ± 1.72 ^c^	7.67 ± 1.63 ^d^	7.00 ± 1.60 ^d^	157.87	<0.001 ***
MST (cm)	1.89 ± 0.08 ^a^	2.00 ± 0.09 ^b^	2.67 ± 0.22 ^c^	3.07 ± 0.23 ^d^	3.22 ± 0.25 ^e^	286.36	<0.001 ***
**Control Group (Localized rESWT)**							
Outcome	Baseline (W0)	Post-tx (W5)	Follow-up 1 (W8)	Follow-up 2 (W12)	Follow-up 3 (W16)	*F*	*p*
VAS (Score)	7.15 ± 0.57 ^a^	6.34 ± 0.16 ^b^	4.72 ± 0.40 ^c^	2.98 ± 0.91 ^d^	2.38 ± 1.15 ^de^	221.66	<0.001 ***
ODI (Score)	22.7 ± 5.06 ^a^	21.9 ± 2.33 ^a^	15.5 ± 3.46 ^b^	10.3 ± 4.40 ^c^	10.2 ± 4.23 ^c^	102.55	<0.001 ***
MST (cm)	1.81 ± 0.10 ^a^	1.96 ± 0.14 ^b^	2.51 ± 0.19 ^c^	2.96 ± 0.20 ^d^	2.95 ± 0.19 ^d^	332.82	<0.001 ***

Values are mean ± SD. ^a, b, c, d, e^: Different letters within a row indicate significant differences by the Bonferroni post-hoc test (*p* < 0.05). *** *p* < 0.001.

**Table 4:** Longitudinal interaction and main effects from two-way repeated measures ANOVA (Intention-to-Treat, *N* = 30).

Outcome	Source	Sum of Squares	*df*_1_	*df*_2_	Mean Square	*F*	*p*	Partial *η*^2^
VAS (Score)	Time †	521.25	2.08	58.10	251.22	543.66	<0.001 ***	0.95
	Group × Time †	19.47	2.08	58.10	9.38	20.31	<0.001 ***	0.42
	Between Groups	42.35	1	28	42.35	45.30	<0.001 ***	0.62
ODI (Score)	Time †	4172.84	2.03	56.84	2055.61	232.93	<0.001 ***	0.89
	Group × Time †	298.76	2.03	56.84	147.17	16.68	<0.001 ***	0.37
	Between Groups	686.94	1	28	686.94	17.41	<0.001 ***	0.38
MST (cm)	Time †	39.10	2.22	62.18	17.61	606.47	<0.001 ***	0.96
	Group × Time †	0.24	2.22	62.18	0.11	3.75	0.025 *	0.12
	Between Groups	0.67	1	28	0.67	7.11	0.013 *	0.20

* *p* < 0.05, *** *p* < 0.001.   † Mauchly’s test indicated that the assumption of sphericity was violated for all outcomes (*p* < 0.001); thus, degrees of freedom and *p*-values were adjusted using the Greenhouse–Geisser correction (VAS: *ϵ* = 0.519; ODI: *ϵ* = 0.507; MST: *ϵ* = 0.555).

**Table 5:** ANCOVA for follow-up 3 (W 16) MST with pre-MST as a covariate.

Source	Type III Sum of Squares	*df*	Mean Square	*F*	*p*	*η*^2^ (Partial)
Baseline MST (Covariate)	0.239	1	0.239	5.849	0.023	0.178
Group (Effect)	0.226	1	0.226	5.542	0.026 *	0.170
Error	1.103	27	0.041			

* *p* < 0.05.

**Table 6:** Comparison of recurrence rates between groups.

Group	Recurrence	No Recurrence	*p*
Experimental	1 (6.7%)	14 (93.3%)	
Control	2 (13.3%)	13 (86.7%)	1.00

(Fisher’s exact test).

## Data Availability

The datasets generated and analyzed during the current study are available from the corresponding author upon reasonable request.

## Supplementary Materials

The following supporting information can be downloaded at: https://www.mdpi.com/article/10.3390/jcm15218531/s1, Supplementary File S1: Original Study Protocol Summary and Methodological Clarifications; Supplementary File S2: Statistical Syntax and Unedited Output.

## Abbreviations

The following abbreviations are used in this manuscript:

NSLBP	Non-Specific Low Back Pain
rESWT	Radial Extracorporeal Shock Wave Therapy
VAS	Visual Analog Scale
ODI	Oswestry Disability Index
MST	Modified Schober Test
ANCOVA	Analysis of Covariance

## References

[1] (2023). GBD 2021 Low Back Pain Collaborators. Global, regional, and national burden of low back pain, 1990–2020, its attributable risk factors, and projections to 2050: A systematic analysis of the Global Burden of Disease Study 2021. Lancet Rheumatol..

[2] Balagué F., Mannion A.F., Pellisé F., Cedraschi C. (2012). Non-specific low back pain. Lancet.

[3] Maher C., Underwood M., Buchbinder R. (2017). Non-specific low back pain. Lancet.

[4] Qaseem A., Wilt T.J., McLean R.M., Forciea M.A. (2017). Noninvasive Treatments for Acute, Subacute, and Chronic Low Back Pain: A Clinical Practice Guideline From the American College of Physicians. Ann. Intern. Med..

[5] Foster N.E., Anema J.R., Cherkin D., Chou R., Cohen S.P., Gross D.P., Ferreira P.H., Fritz J.M., Koes B.W., Peul W. (2018). Prevention and treatment of low back pain: Evidence, challenges, and promising directions. Lancet.

[6] Yue L., Sun M.S., Chen H., Mu G.-Z., Sun H.-L. (2021). Extracorporeal shockwave therapy for treating chronic low back pain: A systematic review and meta-analysis of randomized controlled trials. BioMed Res. Int..

[7] Maffulli N., Maffulli G.D. (2018). The assessment of the effectiveness of extracorporeal shock wave therapy (ESWT) for soft tissue injuries (ASSERT): The future. Muscles Ligaments Tendons J..

[8] Notarnicola A., Maccagnano G., Gallone M., Mastromauro L., Rifino F., Pesce V., Covelli I., Moretti B. (2018). Extracorporeal shockwave therapy versus exercise program in patients with low back pain: Short-term results of a randomized controlled trial. J. Biol. Regul. Homeost. Agents.

[9] Walewicz K., Taradaj J., Rajfur K., Ptaszkowski K., Kuszewski M.T., Sopel M., Dymarek R. (2019). The effectiveness of radial extracorporeal shock wave therapy in patients with chronic low back pain: A prospective, randomized, single-blinded pilot study. Clin. Interv. Aging.

[10] Guo X., Li L., Yan Z., Li Y., Peng Z., Yang Y., Zhang Y., Schmitz C., Feng Z. (2021). Efficacy and safety of treating chronic nonspecific low back pain with radial extracorporeal shock wave therapy (rESWT), rESWT combined with celecoxib and eperisone (C + E) or C + E alone: A prospective randomized trial. J. Orthop. Surg. Res..

[11] Casato G., Stecco C., Busin R. (2019). Role of fasciae in nonspecific low back pain. Eur. J. Transl. Myol..

[12] Stecco A., Macchi V., Stecco C., Porzionato A., Day J.A., Delmas V., De Caro R. (2009). Anatomical study of myofascial continuity in the anterior region of the upper limb. J. Bodyw. Mov. Ther..

[13] Myers T.W. (2020). Anatomy Trains: Myofascial Meridians for Manual and Movement Therapists.

[14] Wilke J., Krause F. (2019). Myofascial chains of the upper limb: A systematic review of anatomical studies. Clin. Anat..

[15] Khan A.A., Srivastava A., Passi D., Devi M., Chandra L., Atri M. (2018). Management of myofascial pain dysfunction syndrome with meditation and yoga: Healing through natural therapy. Natl. J. Maxillofac. Surg..

[16] World Medical Association (2013). World Medical Association Declaration of Helsinki: Ethical principles for medical research involving human subjects. JAMA.

[17] Gleitz M. (2019). Myofascial Syndromes and Trigger Points: Radial Shock Wave Therapy.

[18] Bordoni B., Myers T. (2020). A review of the theoretical fascial models: Biotensegrity, fascintegrity, and myofascial chains. Cureus.

[19] Stecco C., Tiengo C., Stecco A., Porzionato A., Macchi V., Stern R., De Caro R. (2013). Fascia redefined: Anatomical features and technical relevance in fascial flap surgery. Surg. Radiol. Anat..

[20] Wilke J., Krause F., Vogt L., Banzer W. (2016). What is evidence-based about myofascial chains: A systematic review. Arch. Phys. Med. Rehabil..

[21] Langevin H.M., Fox J.R., Koptiuch C., Badger G.J., Naumann A.C.G., Bouffard N.A., Konofagou E.E., Lee W.-N., Triano J.J., Henry S.M. (2011). Reduced thoracolumbar fascia shear strain in human chronic low back pain. BMC Musculoskelet. Disord..

[22] Schleip R., Stecco C., Driscoll M., Huijing P. (2021). Fascia: The Tensional Network of the Human Body.

[23] Ostelo R.W., Deyo R.A., Stratford P., Waddell G., Croft P., Von Korff M., Bouter L.M., de Vet H.C. (2008). Interpreting change scores for pain and functional status in low back pain: Towards international consensus regarding minimal important change. Spine.

[24] Lachin J.M. (2016). Fallacies of last observation carried forward analyses. Clin. Trials.

[25] Vos T., Flaxman A.D., Naghavi M., Lozano R., Michaud C., Ezzati M., Shibuya K., Salomon J.A., Abdalla S., Aboyans V. (2012). Years lived with disability for 1160 sequelae of 289 diseases and injuries 1990–2010: A systematic analysis for the Global Burden of Disease Study 2010. Lancet.

[26] Hodges P.W., Smeets R.J. (2015). Interaction between pain, movement, and physical activity. Clin. J. Pain.

[27] Speed C. (2014). A systematic review of shockwave therapies in soft tissue conditions: Focusing on the evidence. Br. J. Sports Med..

[28] Hoy D., Bain C., Williams G., March L., Brooks P., Blyth F., Woolf A., Vos T., Buchbinder R. (2012). A systematic review of the global prevalence of low back pain. Arthritis Rheum..

[29] Burk C., Perry J., Lis S., Dischiavi S., Bleakley C. (2019). Can myofascial interventions have a remote effect on ROM? A Systematic Review and Meta-Analysis. J. Sport Rehabil..

[30] Van Middelkoop M., Rubinstein S.M., Verhagen A.P., Ostelo R.W., Koes B.W., Van Tulder M.W. (2010). Exercise therapy for chronic nonspecific low-back pain. Best Pract. Res. Clin. Rheumatol..

