# Supplementary material for: Effects of Myofascial Chain-Based Radial Extracorporeal Shock Wave Therapy in Chronic Non-Specific Low Back Pain: A Randomized Controlled Trial
Source: J Clin Med. 2026 Nov 2;15(21):8531. doi: 10.3390/jcm15218531 (PMC13763949; doi:10.3390/jcm15218531)
Supplement: Supplementary file 1 [file jcm-15-08531-s001.zip › Supplementary File S2.pdf]

Supplementary File S2: Statistical Syntax and Output

Manuscript ID: jcm-4582619

Title: Effects of Myofascial Chain-Based Radial Extracorporeal Shock Wave Therapy in Chronic Non-Specific Low Back Pain: A Randomized Controlled Trial

This supplementary document provides the exact statistical software syntax and the corresponding critical output tables used to generate the primary endpoint contrasts (VAS and ODI) via the Repeated-Measures General Linear Model (GLM). The analysis is based on the Intention-to-Treat (ITT) population ($N = 30$) utilizing Last Observation Carried Forward (LOCF) for missing longitudinal data. All analyses were executed using IBM SPSS Statistics for Windows, Version 22.0.

Part 1. SPSS Syntax Code

The following syntax was utilized to run the two-way repeated-measures ANOVA, specifying 'Group' as the between-subjects factor, 'Time' as the 5-level within-subjects factor, and extracting the estimated marginal means with Bonferroni adjustments for pairwise between-group comparisons at each specific time point.

1.1. Syntax for Visual Analog Scale (VAS)

```
GLM VAS_Baseline VAS_Week5 VAS_Week8 VAS_Week12 VAS_Week16 BY Group
  /WSFACTOR=Time 5 Polynomial
  /METHOD=SSTYPE(3)
  /EMMEANS=TABLES(Group*Time) COMPARE(Group) ADJ(BONFERRONI)
  /PRINT=DESCRIPTIVE ETASQ HOMOGENEITY
  /CRITERIA=ALPHA(.05)
  /WSDESIGN=Time
  /DESIGN=Group.
```

1.2. Syntax for Oswestry Disability Index (ODI)

```
GLM ODI_Baseline ODI_Week5 ODI_Week8 ODI_Week12 ODI_Week16 BY Group
  /WSFACTOR=Time 5 Polynomial
```

```
/METHOD=SSTYPE(3)
/EMMEANS=TABLES(Group*Time) COMPARE(Group) ADJ(BONFERRONI)
/PRINT=DESCRIPTIVE ETASQ HOMOGENEITY
/CRITERIA=ALPHA(.05)
/WSDESIGN=Time
/DESIGN=Group.
```

Part 2. Primary SPSS Output: Visual Analog Scale (VAS)

2.1. Tests of Within-Subjects Effects (VAS)

(Mauchly's Test of Sphericity: $p < 0.001$; Greenhouse-Geisser Epsilon = 0.519)

Source	Type III Sum of Squares	df	Mean Square	F	Sig. (p-value)	Partial Eta Squared (ηp2)
**Time** (Greenhouse-Geisser)	521.248	2.075	251.224	543.662	**< 0.001**	0.951
**Time * Group** (Greenhouse-Geisser)	19.470	2.075	9.384	20.308	**< 0.001**	0.420
**Error (Time)** (Greenhouse-Geisser)	26.846	58.095	0.462			

2.2. Estimated Marginal Means: Group * Time (VAS)

Group	Time Point	Mean	Std. Error	95% Confidence Interval (Lower)	95% Confidence Interval (Upper)
**Experimental**	1 (Baseline)	7.120	0.151	6.811	7.429
	2 (Week 5)	4.293	0.087	4.115	4.472
	3 (Week 8)	3.047	0.096	2.849	3.244
	4 (Week 12)	2.067	0.183	1.692	2.442
	**5 (Week 16)**	**1.733**	**0.231**	1.261	2.206
**Control**	1 (Baseline)	7.153	0.151	6.845	7.462
	2 (Week 5)	6.340	0.087	6.162	6.518
	3 (Week 8)	4.720	0.096	4.523	4.917
	4 (Week 12)	2.980	0.183	2.605	3.355
	**5 (Week 16)**	**2.380**	**0.231**	1.908	2.852

2.3. Pairwise Comparisons Between Groups at Primary Endpoint (VAS)

Based on estimated marginal means. Adjustment for multiple comparisons: Bonferroni.

Time Point	(I) Group	(J) Group	Mean Difference (I-J)	Std. Error	Sig. (p-value)	95% Confidence Interval for Difference (Lower)	95% Confidence Interval for Difference (Upper)
**5 (Week 16)**	Experimental	Control	**-0.647***	**0.245**	**0.014**	**-1.16**	**-0.14**

Note: While the pooled-variance omnibus model contrast above yields p = 0.014 (95% CI [−1.16 to −0.14]), the conservative standalone two-sample t-test at Week 16 (reported in the main text per editorial review) yields p = 0.057 (95% CI [−1.31 to 0.02]).

Part 3. Primary SPSS Output: Oswestry Disability Index (ODI)

3.1. Tests of Within-Subjects Effects (ODI)

(Mauchly's Test of Sphericity: $p < 0.001$*; Greenhouse-Geisser Epsilon = 0.507)*

Source	Type III Sum of Squares	df	Mean Square	F	Sig. (p-value)	Partial Eta Squared (ηp2)
**Time** (Greenhouse-Geisser)	4172.840	2.030	2055.605	232.934	< **0.001**	0.893
**Time * Group**	298.760	2.030	147.174	16.677	<	0.373

Source	Type III Sum of Squares	df	Mean Square	F	Sig. (p-value)	Partial Eta Squared (ηp2)
(Greenhouse-Geisser)					**0.001**	
**Error (Time)** (Greenhouse-Geisser)	501.600	56.839	8.825			

3.2. Estimated Marginal Means: Group * Time (ODI)

Group	Time Point	Mean	Std. Error	95% Confidence Interval (Lower)	95% Confidence Interval (Upper)
**Experimental**	1 (Baseline)	22.000	1.245	19.450	24.550
	2 (Week 5)	13.133	0.601	11.903	14.363
	3 (Week 8)	9.400	0.706	7.954	10.846
	4 (Week 12)	7.667	0.857	5.912	9.422
	**5 (Week**	**7.000**	**0.826**	5.308	8.692

Group	Time Point	Mean	Std. Error	95% Confidence Interval (Lower)	95% Confidence Interval (Upper)
	**16)**				
**Control**	1 (Baseline)	22.733	1.245	20.183	25.283
	2 (Week 5)	21.867	0.601	20.637	23.097
	3 (Week 8)	15.533	0.706	14.087	16.979
	4 (Week 12)	10.267	0.857	8.512	12.022
	**5 (Week 16)**	**10.200**	**0.826**	8.508	11.892

3.3. Pairwise Comparisons Between Groups at Primary Endpoint (ODI)

Based on estimated marginal means. Adjustment for multiple comparisons: Bonferroni.

Time Point	(I) Group	(J) Group	Mean Difference (I-J)	Std. Error	Sig. (p-value)	95% Confidence Interval for Difference (Lower)	95% Confidence Interval for Difference (Upper)
**5 (Week 16)**	Experimental	Control	**-3.200***	**1.168**	**0.011**	**-5.62**	**-0.78**

Part 4. Sensitivity Analysis (BOCF): SPSS Syntax Code

To evaluate the robustness of the primary endpoint against outcome-dependent missingness, a worst-case Baseline Observation Carried Forward (BOCF) sensitivity analysis was conducted. The Week 16 data for the 3 participants who discontinued due to recurrence were replaced with their respective Baseline (Week 0) scores.

4.1. Syntax for BOCF (VAS & ODI)

```
GLM VAS_Baseline VAS_Week5 VAS_Week8 VAS_Week12 VAS_BOCF_W16 BY Group
  /WSFACTOR=Time 5 Polynomial
  /METHOD=SSTYPE(3)
  /EMMEANS=TABLES(Group*Time) COMPARE(Group) ADJ(BONFERRONI)
  /PRINT=DESCRIPTIVE ETASQ
  /CRITERIA=ALPHA(.05)
  /WSDESIGN=Time
  /DESIGN=Group.
```

```
GLM ODI_Baseline ODI_Week5 ODI_Week8 ODI_Week12 ODI_BOCF_W16 BY Group
  /WSFACTOR=Time 5 Polynomial
  /METHOD=SSTYPE(3)
  /EMMEANS=TABLES(Group*Time) COMPARE(Group) ADJ(BONFERRONI)
  /PRINT=DESCRIPTIVE ETASQ
  /CRITERIA=ALPHA(.05)
  /WSDESIGN=Time
  /DESIGN=Group.
```

Part 5. Sensitivity Analysis (BOCF): Pairwise Comparisons at Primary Endpoint

Based on estimated marginal means using the pooled residual error variance. Adjustment for multiple comparisons: Bonferroni.

5.1. BOCF Pairwise Comparison for VAS at Week 16

Time Point	(I) Group	(J) Group	Mean Difference (I-J)	Std. Error	Sig. (p-value)	95% Confidence Interval for Difference (Lower)	95% Confidence Interval for Difference (Upper)
**5 (Week 16)**	Experimental	Control	**-0.320**	**0.576**	**0.583**	**-1.500**	**0.860**

5.2. BOCF Pairwise Comparison for ODI at Week 16

Time Point	(I) Group	(J) Group	Mean Difference (I-J)	Std. Error	Sig. (p-value)	95% Confidence Interval for Difference (Lower)	95% Confidence Interval for Difference (Upper)
**5 (Week 16)**	Experimental	Control	**-2.167**	**1.947**	**0.275**	**-6.155**	**1.821**
