# Supplementary material for: Effects of Myofascial Chain-Based Radial Extracorporeal Shock Wave Therapy in Chronic Non-Specific Low Back Pain: A Randomized Controlled Trial
Source: J Clin Med. 2026 Nov 2;15(21):8531. doi: 10.3390/jcm15218531 (PMC13763949; doi:10.3390/jcm15218531)
Supplement: Supplementary file 1 [file jcm-15-08531-s001.zip › Supplementary_File_S1.pdf]

Supplementary File S1: Original Study Protocol Summary and Methodological Clarifications

Manuscript ID: jcm-4582619

Article Title: Effects of Myofascial Chain-Based Radial Extracorporeal Shock Wave Therapy in Chronic Non-Specific Low Back Pain: A Randomized Controlled Trial

Journal: Journal of Clinical Medicine

Part 1. Trial Overview and Ethics Approval

• Original Protocol Title: Effects of Radial Extracorporeal Shock Wave Therapy Using the Myofascial Chain Concept in Non-Specific Low Back Pain

• Institutional Review Board (IRB) Approval Number: SYU IRB 2023-04-009-007

• IRB Approving Body: Institutional Bioethics Committee of Sahmyook University, Seoul, Republic of Korea

• Original Protocol Version and Submission Date: Version 1.3, submitted on April 19, 2023; ethics approved for implementation through May 14, 2026

• Trial Registry: Clinical Research Information Service (CRIS, Republic of Korea; KCT0011599)

• Principal Investigator (PI): InSeong Choi, PT, PhD Candidate (Department of Physical Therapy, Graduate School, Sahmyook University)

• Sponsoring / Academic Supervisor: JongEun Yim, PT, PhD (Department of Physical Therapy, Graduate School, Sahmyook University)

Part 2. Pre-specified Study Design and Methodological Summary

Below is an English summary of the original study protocol formally approved by the IRB in 2023, prior to participant recruitment and trial commencement:

1. Study Design: A prospective, single-center, randomized controlled trial (1:1 parallel-group allocation).

2. Participants and Eligibility:

• Inclusion Criteria: Adults aged 18-70 years with chronic non-specific low back pain (NSLBP) persisting for 3 months; radiographically confirmed exclusion of lumbar vertebral fracture, spondylolysis, spondylolisthesis, or severe osteoporosis; MRI showing normal findings or mild, asymptomatic disc desiccation/bulging without neurological compromise.

• Exclusion Criteria: Age <18 or 70 years; history of spinal surgery or fracture; specific spinal pathology (acute/symptomatic disc protrusion with radiculopathy, ankylosing spondylitis, structural scoliosis); contraindications to rESWT (pregnancy, coagulopathy, malignancy, local infection, anticoagulant therapy, or corticosteroid injection within 6 weeks).

3. Randomization and Allocation Concealment:

• Sequence generated via computer program (www.random.org).

• Allocation managed via sequentially numbered, opaque, sealed envelopes.

4. Interventions:

• Experimental Group (Myofascial Chain): rESWT applied along interconnected myofascial pathways: origin of gluteus maximus (1,000 pulses), gluteus medius within the hip triangle (1,000 pulses), and distal/mid iliotibial band (1,000 pulses), totaling 3,000 pulses/session (1.0 bar, 5-7 Hz) once weekly for 4 weeks.

• Control Group (Localized): rESWT applied to the two primary lumbosacral pain sites (1,500 pulses per site), totaling 3,000 pulses/session (1.0 bar, 5-7 Hz) once weekly for 4 weeks.

• Common Conservative Care: Identical basic physical therapy (superficial heat, continuous ultrasound, TENS) and home stabilization exercise education.

5. Sample Size Determination: Repeated-measures ANOVA (within-between interaction) calculated using G*Power (3.1.9.7): effect size $f=0.40$, $\alpha= 0.05$, power $(1-\beta)=0.80$, requiring N=24 completers (12 per group); adjusted to N=30 to accommodate an anticipated 20% attrition rate.

6. Pre-specified Follow-up Timeline: Baseline (Week 0), 1-week post-treatment (Week 5), 4-weeks post-treatment (Week 8), 1-month follow-up (Week 12), and 3-month follow-up (Week 16).

Part 3. Methodological Clarifications for Editorial and Peer Review

To facilitate editorial review and reconcile any linguistic or procedural nuances between the original IRB protocol (2023) and the final publication manuscript, the authors provide the following explicit clarifications:

1. Pre-specified Outcomes and Omission of Laitinen Pain Scale (LPS)

In the original IRB protocol approved in 2023 (Sections 12 and 13), both the Visual Analog Scale (VAS) and the exploratory Laitinen Pain Scale (LPS) were listed for pain evaluation. However, during the pre-trial review immediately prior to the commencement of actual participant enrollment in June 2025, a definitive clinical decision was made by the steering committee to eliminate the LPS from the Case Report Forms (CRFs). This decision was made to minimize survey fatigue for the participants and to strictly align the endpoints with internationally

recognized core outcome sets for low back pain, which prioritize the continuous 10-cm VAS. Consequently, LPS data were never systematically collected or recorded for the 30 participants randomized in this trial. The 10-cm VAS and the ODI were exclusively maintained as the primary endpoints.

2. Protocol Dropout Criteria vs. Intention-to-Treat (ITT) Framework

In Section 17 of the original protocol ("Dropout criteria"), it was prospectively specified that: "Recurrence of low back pain symptoms requiring analgesic intervention prior to post-assessment" warranted clinical withdrawal from ongoing study follow-up to allow appropriate medical rescue therapy.

• During the follow-up period, 3 participants (1 experimental, 2 control) reached this clinical withdrawal criterion due to symptom recurrence and discontinued trial follow-up visits.

Methodological Reconciliation: In strict adherence to peer-review recommendations and CONSORT guidelines, these 3 participants were not excluded from our primary efficacy evaluation. Instead, they were analyzed within their originally allocated groups under the Intention-to-Treat (ITT) principle (N=30). Both Last Observation Carried Forward (LOCF) and worst-case Baseline Observation Carried Forward (BOCF) imputation strategies were employed.

Clarification on Consent (Reference to Section 5 of the Patient Information Sheet): Section 5 of the informed consent document states that collected data will be destroyed if a participant formally withdraws consent. However, the 3 participants who experienced recurrence did not voluntarily withdraw their consent. Rather, they met the clinical dropout criteria (Section 17) requiring external medical rescue care, which necessitated the cessation of physical study visits. They explicitly permitted the retention of their previously collected data, allowing for their proper inclusion in the ITT analysis.

3. Systematic Monitoring of Harms and Adverse Events

Section 16 of the original protocol established a prospective monitoring protocol for procedure-related harms: "Procedure-related pain, erythema, and excessive muscle guarding are monitored in real time... supervised directly by the PI and two rehabilitation physicians".

Through this active surveillance system, zero treatment-related adverse events (e.g., severe hematoma, persistent skin lesion, or neurological deterioration) occurred during the trial. Symptom recurrence was evaluated separately as an exploratory clinical efficacy endpoint rather than an adverse intervention harm.

4. Clarification of Blinding and Personnel Roles

In Section 7 of the original document, phrasing regarding investigator masking contained a linguistic ambiguity. In clinical execution:

- Researcher A: Independent sequence generator and keeper of opaque allocation envelopes.
- Therapist B: Designated licensed physical therapist who opened envelopes and delivered the rESWT interventions (unblinded to anatomical site).
- Assessor C: Dedicated outcome assessor and data analyst who remained strictly masked to group allocation throughout the entire study duration.
- Participants: Blinded to study hypotheses and group allocation; both arms received active rESWT with identical acoustic sensations, gel, and prone positioning.
